# Anencephaly alters renal parenchymal volume in human fetuses?

**DOI:** 10.1590/S1677-5538.IBJU.2020.06.02

**Published:** 2020-09-02

**Authors:** Andre L. Lima Diniz, Francisco J. B. Sampaio, Luciano A Favorito

**Affiliations:** 1 Unidade de Pesquisa Urogenital Universidade Estadual do Rio de Janeiro Uerj Rio de JaneiroRJ Brasil Unidade de Pesquisa Urogenital - Universidade Estadual do Rio de Janeiro Uerj, Rio de Janeiro, RJ, Brasil

## INTRODUCTION

The development of ultrasonography in the 1970s enabled the pre-natal diagnosis of fetal malformations during regular monitoring of pregnant women. Birth defects are the leading cause of infant mortality, and in 2013 these were associated with 4.778 deaths (20% of deaths in the first year of life) in United States ( 1 ). In a recent five-year cohort study, researchers reported a prevalence of 2% of major birth defects with estimate of known etiology in just over 20% of these cases ( 2 ).

Although congenital heart diseases are the most common birth defects and the leading cause of infant death worldwide ( 3 ) recently neural tube defects (NTDs) have gained space in scientific production and news reports around the world. Since its introduction in the Americas in 2015, the mosquito-borne Zika-virus (family Flaviviridae) has spread rapidly and has been associated with the increased incidence of microcephaly and anencephaly cases ( 4 - 7 ).

Anencephaly is the most severe fetal NTD, resulting from failure of the neural tube to close at the base of the skull in the third or fourth week (day 26 to 28) after conception, leaving the skull bones that usually surround the head unformed ( 8 ). Anencephaly is observed in 0.03% of all births. It occurs at a rate three to four times higher in female fetuses compared to males ( 8 ).

Due to the obvious diagnosis and prognosis, much discussion has occurred in the last 40 years about organ donation from anencephalic newborns ( 9 - 12 ). Some studies have been carried out to understand the feasibility of using these organs and tissues.

Carvalho et al. ( 13 ) reported no differences in the structure of the genitalia of anencephalic fetuses compared to normal ones. Pazos et al. ( 14 ) found that the bladders of anencephalic fetuses had gross histological alterations when compared to those of normal fetuses. Costa et al. ( 15 ) demonstrated differences in the structure of the ureter. Kalaycioglu et al. ( 16 ) carried out a stereological evaluation of the kidneys of anencephalic and normal fetuses and found no significant differences between the groups.

To our knowledge there are no reports comparing kidney volumetry between normal and anencephalic fetuses. Our hypothesis was that the anencephaly alters the volume of the fetal kidney during the human fetal period. The objective of the study was to analyze the kidney volumetry in anencephalic fetuses according to gender and laterality and compare it to the parameters of normal fetuses.

## MATERIALS AND METHODS

This study was carried out in accordance with the ethical standards of the hospital’s institutional committee on human experimentation. (IRB: 2.079.618, CAAE: 67944217.3.0000.5259).

We studied 102 fetuses: 18 anencephalic (8 females, 10 males), and 84 fetuses without apparent macroscopic malformations (40 females, 44 males), for a total of 204 renal units evaluated, during the period from January 2016 through July 2019. The fetuses came to our laboratory as a donation of the Obstetric section of our hospital. The fetuses of control group were macroscopically well preserved, showed no signs of malformations and the demise is hypoxia. The gestational age was determined in WPC according to the foot-length criterion. This criterion is currently considered the most acceptable parameter to estimate gestational age ( 17 - 19 ). The fetuses were also evaluated regarding crown-rump length (CRL) and body weight immediately before dissection. The same observer made all the measurements. All the kidneys with anomalies (vascular anomalies, fusion, rotation, duplication) and renal pelvis dilation were excluded from the study.

**Figure 1:**
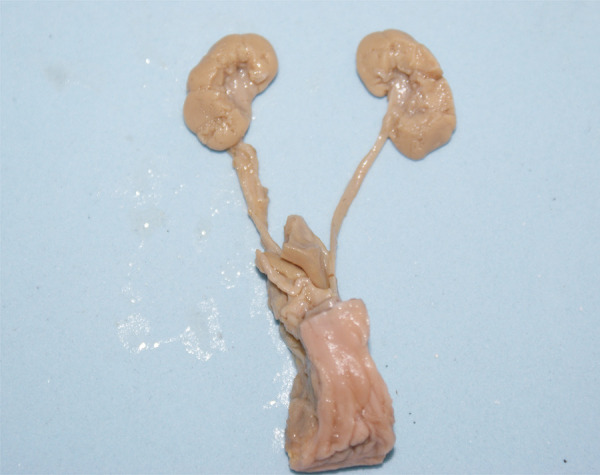
The fetuses were carefully dissected with the aid of a stereoscopic lens with 16/25X magnification and the kidneys were removed together with the ureters, bladder and genital organs before the measurements of renal volumes.

After the measurements, the fetuses were carefully dissected with the aid of a stereoscopic lens with 16/25X magnification. The kidneys were removed together with the ureters, bladder and genital organs ( Figure-1 ). After kidney dissection, we evaluated the following measurements: renal length, width of the superior pole, width of the inferior pole, and renal thickness.

After obtaining the kidney measurements, we carefully dissected the renal pelvis and the major calyces, with removal of the renal parenchyma around the renal pelvis, whenever necessary, for accurate identification and measurement of these structures. The following renal pelvis measurements were taken with the help of a magnifying lens and a digital pachymeter (calibrated in millimeters): transverse diameter of the renal pelvis (measurement obtained between the distal pelvis extremity and the confluence of the major calyces), and the longitudinal diameter of the renal pelvis (distance between the two extremities of the pelvis, i.e., upper-most and lower-most).

The fetal renal volume was calculated using the ellipsoid formula (10): Renal volume (RV)=[renal length x renal thickness x renal width (lower pole+upper pole)/2] x 0.523. We considered the thickness of the renal pelvis as 1mm, so the fetal renal pelvis volume was calculated using the ellipsoid formula too: fetal renal pelvis volume (RpvV)=renal pelvis length x renal pelvis thickness (=1mm) x renal pelvis width x 0.523. An accurate assessment of the renal parenchymal volume (RpcV) can be obtained by subtacting the volume of the renal pelvis from the total renal volume ( 20 - 24 ).

### Statistical analysis

All parameters were statistically processed and graphically described. The Shapiro-Wilk test was employed to ascertain the normality of the data and to compare quantitative data between normal vs. anencephalic fetuses, while the Kruskal-Wallis test was used to assess gender and laterality differences. Simple linear correlations (LC) were calculated for total renal volume and renal parenchymal volume according to fetal age, weight and crown-rump length. The statistical analysis was performed with the RStudio program (Version 1.0.143).

## RESULTS

The fetuses presented gestational ages between 12 and 23 WPC (median=17WPC; IQR=2.75); weigh between 30 and 780g (median=227.5g; IQR=145.75); and had crown-rump length between 9.5 and 22.2cm (median=15.5cm; IQR=3). The summary of the findings regarding the total renal volume (RV), renal pelvis (RPvV) and renal parenchyma (RPcV) is shown in Table-1 .

**Table 1:** The table shows the parameters of renal measurements of the human fetuses analyzed.

Measurements	Female Fetuses										Male Fetuses								

	Right					Left					Right					Left			

	Normal		Anencephalic		Normal			Anencephalic		Normal			Anencephalic		Normal			Anencephalic	

	$\tilde{X}$	IQR	$\tilde{X}$	IQR	$\tilde{X}$		IQR	$\tilde{X}$	IQR	$\tilde{X}$		IQR	$\tilde{X}$	IQR	$\tilde{X}$		IQR	$\tilde{X}$	IQR
RV (mm^3^)	740.00	609.74	940.00	241.30	787.00		607.50	940.30	183.81	655.00		546.50	582.00	214.30	591.00		432.50	555.00	306.83
RPcV (mm^3^)	736.85	607.20	935.90	230.03	779.02		607.73	930.50	177.12	647.60		542.65	576.06	212.35	585.35		419.00	542.20	303.20
RPvV (mm^3^)	8.64	4.70	7.84	7.66	8.53		4.11	6.86	5.00	8.52		7.45	7.82	8.55	8.70		7.75	8.90	3.31

**RV** = Renal volume (mm^3^); **RPvV** = Renal pelvis volume(mm^3^); **RPcV** = Renal parenchyma volume(mm^3^); = Median; **IQR** = interquartile range

Concerning the whole sample, the median RV of the right kidney was 676.1mm^3^(IQR=598.46) and left kidney was 620.8mm^3^(IQR=544). No statistical difference was observed between the sides (p=0.31). No statistical difference was observed between genders for right kidney (p=0.06) or for left kidney (p=0.049).

Concerning the whole sample, the median RPcV of the right kidney was 666.22mm^3^(IQR=595.83) and left kidney was 606.76 mm^3^(IQR=539.21). No statistical difference was observed between the sides (p-value=0.48) and no statistical difference was observed between genders for the right kidney (p=0.06), but a statistical difference was observed between genders for the left kidney (p=0.046).

Also regarding the whole sample, the median RPvV of the right kidney was 8.40mm^3^ (IQR=6.50) and left kidney was 8.61mm^3^(IQR=5.67). No statistical difference was observed between the sides (p=0.41), and no statistical difference was observed between genders for right kidney (p=0.58) or for left kidney (p=0.71).

Disregarding the gender, when comparing the differences in renal volumetry between anencephalic fetuses and normal fetuses, there were no statistically significant differences of RV for right kidney (p=0.62), and left kidney (p=0.97). RPcV also was not statistically different for right kidney (p=0.60) and left kidney (p=0.95), nor was RPvV for right kidney (p=0.33) and left kidney (p=0.77).

Comparing anencephalic male fetuses to normal male fetuses, we found no statistical difference for the right kidney regarding RV (p=0.09), RPcV (p=0.09) and RPvV (p=0.95), while for the left side, no significant statistical difference was observed regarding RV (p=0.13), RPcV (p=0.12) and RPvV (p=1.0).

Comparing female anencephalic fetuses to female normal fetuses, we found no statistical difference for the right kidney regarding RV (p=0.31), RPcV (p=0.32) and RPvV (p=0.23), while for the left side, no significant statistical difference was observed regarding RV (p=0.14), RPcV (p=0.14) and RPvV (p=0.61).

**Figure 2:**
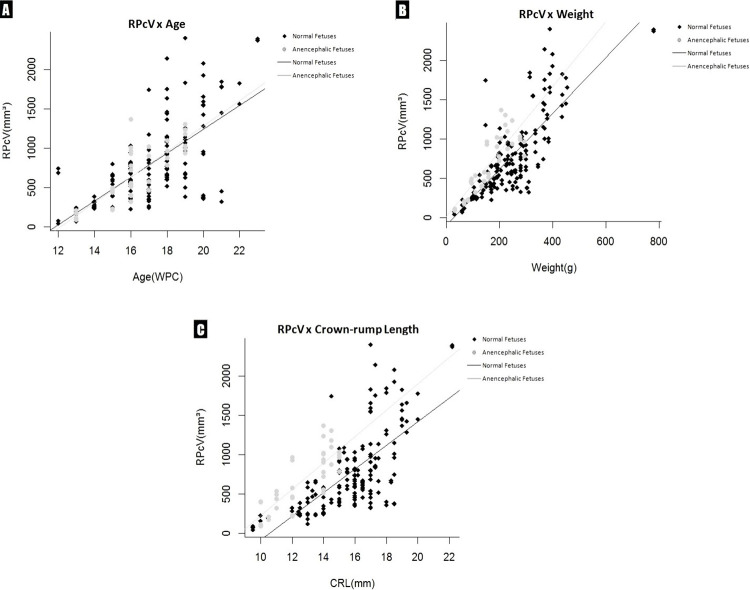
Correlation of the renal parenchymal volume (RPcV) for normal and anencephalic fetuses with fetal age, crown-rump length (CRL) and weight during the fetal period studied. The points plotted represent the mean values obtained for each week studied. A) AGE (WPC). Linear regression indicated that RPcV is correlated significantly and positively with fetal age (normal fetuses: r2 = 0.48, and anencephalic fetuses: r2 =0.64, p <0.0001). B) Fetal weight (g). Linear regression indicated that RPcV is correlated significantly and positively with fetal weight (normal fetuses: r2 = 0.65, and anencephalic fetuses: r2 = 0.71, p <0.0001). C) Crown-rump length (cm). Linear regression indicated that RPcV is correlated significantly and positively with fetal crown-rump length (normal fetuses: r2 = 0.48, and anencephalic fetuses: r2 = 0.64, p <0.0001).

The linear correlation comparing renal parenchymal volume data and fetal anthropometry were assessed ( Figure 2 ). Although all the correlations were positive, it must be said that r^2^ values less than 0.4 reflect very weak correlation, while r^2^ between 0.4 and 0.7 reflect moderate correlation and r^2^ greater than 0.7 indicates strong correlation.

Linear regression indicated that RPcV is correlated significantly and positively with fetal age (normal fetuses: r^2^ = 0.48, and anencephalic fetuses: r^2^ = 0.64, p<0.0001); fetal weight (normal fetuses: r^2^ = 0.65, and anencephalic fetuses: r^2^ = 0.71, p <0.0001) and crown-rump length (normal fetuses: r^2^ = 0.48, and anencephalic fetuses: r^2^ = 0.64, p <0.0001).

## DISCUSSION

Nephrons starts this development in the 8th week and all of the branches of the ureteric bud and the nephron units have been formed by 32 to 36 weeks of gestation however, these structures are not yet mature, and will continue to mature after birth ( 25 ).

Since anencephalic newborns are not viable or tractable and the survival is measured in hours or days ( 26 ), the possibility exists of donating their organs for transplantation. Those in favor of this donation argue it is a way to save lives and give meaning to death. Knowledge of the effects that NTDs can cause to other systems is crucial to ascertain which organs can be donated. This has been done since 1960s, as described by Cabasson and colleagues, who studied the anatomy of hearts in anencephalic newborns ( 9 ).

Recently, Costa et al. ( 27 ) studied 17 human fetuses with NTDs and found that urogenital anomalies in human fetuses with NTDs are significant, with an incidence greater than 20%, but did not find severe urogenital anomalies that impair body function. The same group reported that in fetuses with anencephaly, the ureters had smaller diameter, area and thickness, which could indicate that this tissue tends to have significant structural alterations, probably due to cerebral lesions with consequent brain control damage of ureter nerves ( 15 ).

Deepening the discussion about this, Kalaycioglu et al. ( 28 ) performed studies of anatomical and histological aspects of the kidneys in human fetuses with anencephaly, comparing them with normal fetuses. In their first paper, they analyzed fetuses at gestational ages from 30-35 weeks. They found no statistical macroscopic difference between the two groups terms of kidney width, height, weight and volume, while microscopic assessment found no statistical differences in terms of kidney volume and the number and height of the glomeruli 7. The second paper analyzed fetuses from 25-30 weeks, using the same methods, and reported the same results, suggesting that anencephalic fetuses do not differ from normal fetuses regarding kidneys ( 16 ).

We focused on the macroscopic aspect of the kidneys, evaluating the parenchyma, collecting system and total volume in fetuses in the second trimester of gestation. At this age, the kidneys are expected to have reached their final position and from then on they will only develop in size, representing an ideal moment for this study. This paper is the first in the literature to report the renal parenchyma volume in anencephalic fetuses. We observed that the anencephalic fetuses’ kidneys had almost the same measurements and parenchyma volume as those of normal fetuses. This is very important information and suggests that the kidney development is unchanged by this kind of neural tube defect. Based on our findings we can extrapolate and suggest that the anencephalic kidneys can be used as allografts in a scenario of transplantation, depending on ethical and legal considerations.

We should mention some limitations of this study: First, considering the kidney as being an ellipsoid and using the corresponding formula could underestimate the real volume ( 29 ). Second, the absence of urine in the pelvis led us to use a constant thickness of 1mm. However, while urine volume and pelvic distension/diameter are dynamic measurements that fluctuate when measured with ultrasound, in this anatomical study this standardization was a key to overcome that limitation. So, we considered this to be a reasonable way to achieve results, since it is reproducible. Third, we calculated renal parenchymal volume by subtracting pelvis volume from total kidney volume. If we had considered an extra-renal pelvis, the final arithmetic likely would have shown lower parenchymal volume. Fourth, the sample size was small. The 204 units studied were not adequate to determine kidney growth and make comparisons with sufficient accuracy. However, access to human fetuses is limited, so observations of this sample of 102 human fetuses may be important despite the small sample. Furthermore, there may have been bias in the sample regarding WPC distribution. Nevertheless, the sample distribution during the period of fetal kidney development studied was adequate in our opinion.

## CONCLUSIONS

The analysis indicated that renal volumetry is not affected by anencephaly neural tube defects. The parameters reported here provide a reference for functional renal volumetry, suggesting that kidneys from anencephalic fetuses have the same development as normal fetuses.

## ABBREVIATIONS

NTD: neural tube defect
WPC: weeks post conception
CRL: crown rump length
L: longitudinal length
SW: superior pole width
IW: inferior pole width
T: thickness
RPL: renal pelvis length
RPW: renal pelvis width
RV: renal volume
RPcV: renal parenchymal volume
RPvV: renal pelvis volume
$\tilde{X}$: median
IQR: interquartile range
LC: linear correlation
r^2^: correlation coefficient
